# Real World Lecanemab Outcome Data at 6 months in an Academic Healthcare System

**DOI:** 10.1002/alz70861_108466

**Published:** 2025-12-23

**Authors:** Kelania Jimenez, Thomas J Grabowski, Michael Henry Rosenbloom, Varun Mehta

**Affiliations:** ^1^ University of Washington, Seattle, WA USA; ^2^ Memory and Brain Wellness Center, University of Washington, Seattle, WA USA; ^3^ University of Wisconsin, Madison, WI USA

## Abstract

**Background:**

Monoclonal antibodies targeting amyloid‐beta plaques have emerged as a new disease‐modifying drug class that target and remove cerebral amyloid in early‐stage Alzheimer’s disease. Whereas the CLARITY‐AD trial demonstrated significant cognitive and functional slowing as measured by the CDR‐SB at 18 months, limited data relating to real‐world cognitive and behavioral outcomes exists. Our objective was to describe 6‐month clinical outcomes in a community sample of early‐stage AD subjects receiving lecanemab.

**Method:**

We reviewed retrospective observational data from 79 early stage‐AD patients receiving mABs over 6 months at an academic community hospital‐based clinic in Seattle, WA. All patients were administered the Montreal Cognitive Assessment (MOCA), functional assessment questionnaire (FAQ), patient health questionnaire (PHQ‐9), and generalized anxiety disorder (GAD‐7) as part of usual clinical protocol. Cognitive screening tests performed up to one year prior to lecanemab served as a baseline assessment. Demographics and incidence of amyloid related imaging abnormalities‐edema/microhemorrhage (ARIA‐E) and (ARIA‐H) were calculated.

**Result:**

Average patient age was 71.6 (59.5% female) with 65.8% of patients carrying an MCI diagnosis. Racial demographics showed mostly White patients (94.9%) with limited representation of diverse populations (2.5% unknown; 1.3% Asian; 1.3% Native American; 1.3% Latino ethnicity). The majority of treated patients were ApoE4 carriers (70.8%) with 12.7% of patients being homozygous for ApoE4. Mean MOCA score was 21.1±1.4 at baseline, 19.9±4.2 at 6 months; FAQ score 6.4±2.8 at baseline, 8.1±2.8 at 6 months; PHQ9 score 2.1±0.7 at baseline, 1.5±4.2 at 6 months; GAD‐7 score 2.0±2.1 at baseline, 0.9±1.6 at 6 months. Fifteen patients (19%) developed either ARIA‐E or ARIA‐H. Of those 15 patients, 8 had isolated ARIA‐H (53.3%), 3 had isolated ARIA‐E (20%), and 4 (27%) patients showed mixed findings of both.

**Conclusion:**

In the interim analysis, a trend toward improved depression and anxiety scores over 6 months was observed with modest declines in cognition and performance. A full data set analysis will provide comprehensive data relating to clinical outcomes with mAb treatments. Further analysis of longitudinal data is necessary to confirm whether anti‐amyloid monoclonal antibody clinical trial efficacy translates into real world clinical effectiveness at 12 and 18 months.

